# Plant Polysaccharides in Engineered Pharmaceutical Gels

**DOI:** 10.3390/bioengineering9080376

**Published:** 2022-08-09

**Authors:** Juliana O. Bahú, Lucas R. Melo de Andrade, Raquel de Melo Barbosa, Sara Crivellin, Aline Pioli da Silva, Samuel D. A. Souza, Viktor O. Cárdenas Concha, Patrícia Severino, Eliana B. Souto

**Affiliations:** 1 National Institute of Science and Technology in Biofabrication (INCT-BIOFABRIS), School of Chemical Engineering, University of Campinas, Albert Einstein Ave., Cidade Universitária Zeferino Vaz, Campinas 13083-852, SP, Brazil; 2 Laboratory of Pharmaceutical Technology, Faculty of Pharmaceutical Sciences, Food and Nutrition, Federal University of Mato Grosso do Sul, Campo Grande 79070-900, MS, Brazil; 3 Department of Pharmacy, Federal University of Rio Grande do Norte, Campus Universitário, Lagoa Nova Natal 59078-970, RN, Brazil; 4 Institute of Environmental, Chemical and Pharmaceutical Science, School of Chemical Engineering, Federal University of São Paulo (UNIFESP), São Nicolau St., Jd. Pitangueiras, Diadema 09913-030, SP, Brazil; 5 Laboratory of Nanotechnology and Nanomedicine (LNMed), Institute of Technology and Research (ITP), Murilo Dantas Ave., Farolândia, Aracaju 49032-490, SE, Brazil; 6 Industrial Biotechnology Program, Tiradentes University (UNIT), Murilo Dantas Ave., Farolândia, Aracaju 49032-490, SE, Brazil; 7 Department of Pharmaceutical Technology, Faculty of Pharmacy, University of Porto (FFUP), Rua Jorge de Viterbo Ferreira, 4050-313 Porto, Portugal; 8 REQUIMTE/UCIBIO, Faculty of Pharmacy, University of Porto, de Jorge Viterbo Ferreira, 4050-313 Porto, Portugal

**Keywords:** absorbent, bio-based, drug delivery, gums, hydrogels, lignocellulosic, scaffolds

## Abstract

Hydrogels are a great ally in the pharmaceutical and biomedical areas. They have a three-dimensional polymeric structure that allows the swelling of aqueous fluids, acting as an absorbent, or encapsulating bioactive agents for controlled drug release. Interestingly, plants are a source of biogels, specifically polysaccharides, composed of sugar monomers. The crosslinking of these polymeric chains forms an architecture similar to the extracellular matrix, enhancing the biocompatibility of such materials. Moreover, the rich hydroxyl monomers promote a hydrophilic behavior for these plant-derived polysaccharide gels, enabling their biodegradability and antimicrobial effects. From an economic point of view, such biogels help the circular economy, as a green material can be obtained with a low cost of production. As regards the bio aspect, it is astonishingly attractive since the raw materials (polysaccharides from plants-cellulose, hemicelluloses, lignin, inulin, pectin, starch, guar, and cashew gums, etc.) might be produced sustainably. Such properties make viable the applications of these biogels in contact with the human body, especially incorporating drugs for controlled release. In this context, this review describes some sources of plant-derived polysaccharide gels, their biological function, main methods for extraction, remarkable applications, and properties in the health field.

## 1. Introduction

**Figure 1:**
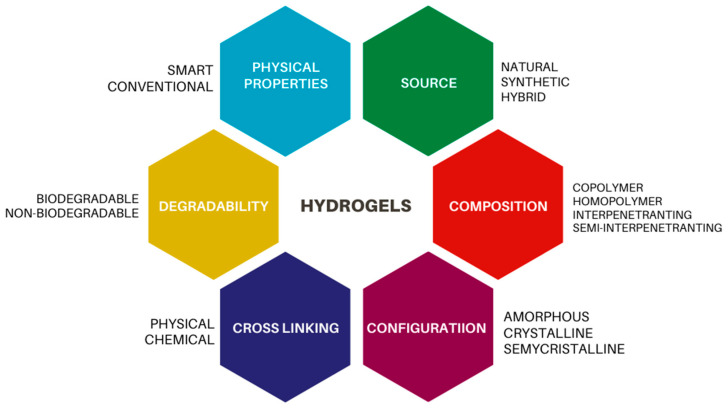
Schematic representation of the hydrogels’ classification (Adapted with permission from Ref. [4]. Copyright 2015, Elsevier).

Hydrogels are defined as a system that can be composed of multiple components (crosslinking agents, monomers, radical initiator) forming a three-dimensional (3D) polymeric network, the structure of which can be filled with water between the macromolecules’ space [1,2]. Their polymeric structure expands when dispersed in water, yielding a stable dispersion thanks to hydrogen bonds. The hydrogel is associated with several variables that mainly include the physical properties, composition, nature of swelling, origin, sources, ionic charges, rate of biodegradation, and observed nature of crosslinking defined its classification [1,3] (Figure 1).

The structure of hydrogels is formed by crosslinking the polymer through non-covalent or covalent interactions, leading them to gelation [5,6,7]. However, some mechanical properties can be predictable, in a way that new functional groups can be introduced into their network, via the addition of functionalized monomers that are compatible with the polymerization procedure [6,8]. The diversity of hydrogels, natural and synthetic, with different chemical compositions and polymer topologies, make them highly adaptable to a wide range of industrial applications such as pharmaceutical, biomedical, cosmetical, coatings, agriculture, contact lens, food packaging, etc. [9].

**Figure 2:**
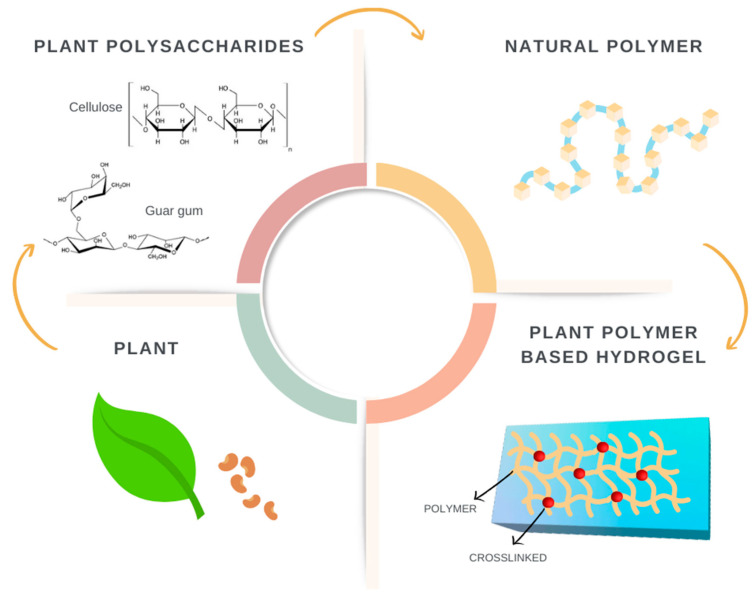
Plant-derived polymers used in hydrogels’ development.

Biopolymers are natural polymers that caught the attention of researchers and society, since they present a sustainable approach to materials development [10]. They are divided into three groups: (i) polysaccharides, (ii) polypeptides, and (iii) polynucleotides. Polysaccharides are the most abundant in nature, used for the development of drug delivery systems, vascular tissue engineering, and biomedical applications [11,12]. The literature reports the use of various hydrogels based on plant-derived polymers, such as cellulose, hemicellulose, lignin, inulin, pectin, starch, guar gum, among others [13], as depicted in Figure 2.

Biotechnology has focused on plant polysaccharides to obtain innovative products to apply them following the bioeconomy principles, for which a set of practices are based on the intelligent use of natural resources, to satisfy current needs while guaranteeing resources for future generations [14]. There is an immense variety of raw materials in nature that can be used in their primary form or used to obtain new materials. For pharmaceutical applications, we have as examples, regenerative medicine, drug delivery, encapsulation of active ingredients; and for cosmetics, hair care, oral care, skincare, and mucous membrane care. The similarity between these three-dimensional polymeric networks with human tissue has potential application in tissue engineering, due to their high biodegradability, low development cost, and low toxicity [15].

In this review, we discuss the latest developments of plant polysaccharide hydrogels, their main material sources, physicochemical properties, and applications that justify their interest in the development of products for pharmaceutical and biomedical applications. Current challenges and future perspectives are also addressed.

## 2. Polymers

The word *polymer* is Greek, where *poly* means many, and *mer* refers to a repeating unit. In this context, *mer* corresponds to a unitary group of atoms or molecules, which defines the polymer characteristic arrangement. Thus, a polymer can be considered the combination of long *mers’* sequences.

Polymers are composed of giant molecules, called macromolecules, formed by the union of monomers through chemical reactions (polymerization). For example, the polymerization of ethylene (ethene), produces polyethylene (PE), which can contain up to 50,000 carbon atoms in one polymeric chain.

**Figure 3:**
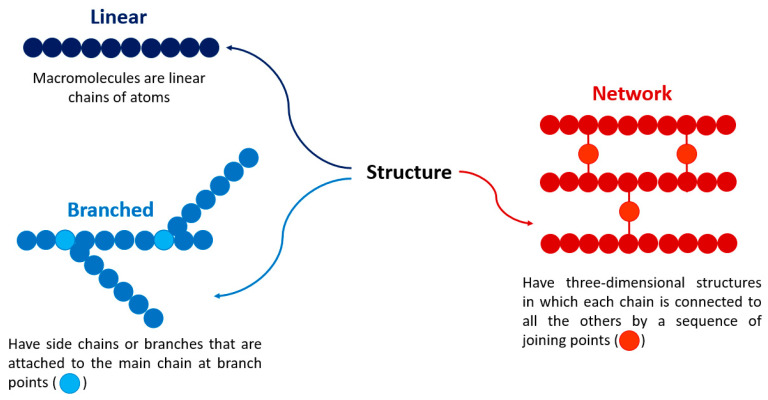
Classification of polymers by structure.

**Figure 4:**
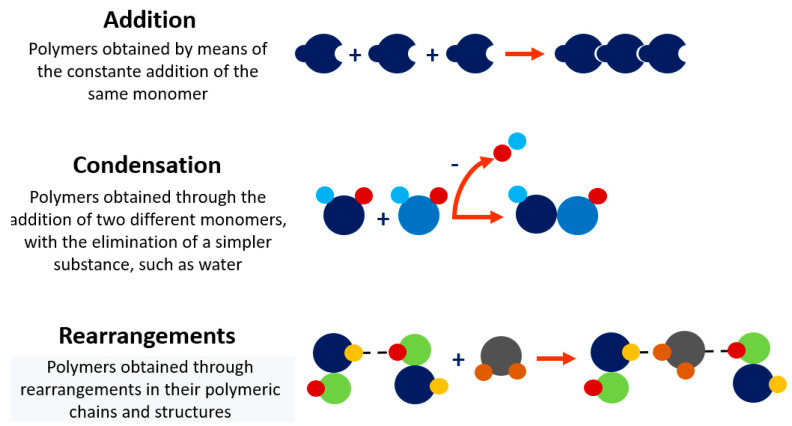
Description and illustration of methods for obtaining polymers.

There are several classifications for polymers, mainly in terms of structure, number of monomers, method of obtaining, and nature. The linear, branched, or network polymers structures are shown in Figure 3. Monomers’ quantity categorizes them as homopolymers and copolymers. Homopolymers are polymers derived from just one type of monomer, while the copolymers chains are formed by more than one type of monomer; both being linear, branched, or crosslinked. Figure 4 illustrates the methods for their synthesis.

Natural polymers are all of those found in nature, for example, rubber, from the tree *Hevea brasiliensis*, which produces isoprene as its main monomer; and polysaccharides (cellulose, starch, glycogen, agar, pectin, aloe vera gel, xanthan gum, alginate, etc.). Synthetic polymers are manufactured and generally contain petroleum-derived ingredients, e.g., polystyrene (PS), polyvinyl chloride (PVC), polyethylene (PE), polypropylene (PP) [16].

## 3. Plant-Derived Polymers

**Figure 5:**
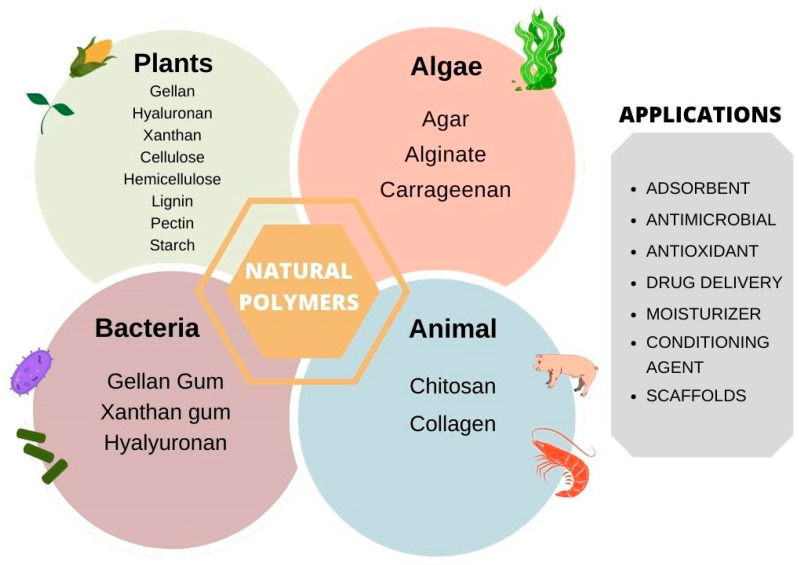
Bio-gels derived from natural sources and their functions.

In recent years, products derived from natural, renewable, and biological-based biodegradable products, including lignocellulosic biomass as raw material, have drawn attention to the development of products for cosmetic, pharmaceutical, and biomedical applications. Bio-gels can also be derived from natural resources, such as animals, plants, algae, and microorganisms, some of which are depicted in Figure 5. These gels have a crosslinked polymer network with hydrophilic characteristics, biodegradability, and biocompatibility that make them suitable for contact with humans. Therefore, their properties involve viscosity enhancer, water swelling, adsorbent, etc., which sustainable architecture of the gel promotes their applications in drug delivery systems [6,17]. It is worth mentioning that plant-derived gels have a GRAS certificate (Generally Recognized as Safe), which enables their use as a material for encapsulating drugs for oral/buccal management and their contact with the human body as scaffolds for tissue engineering [18].

However, plant-derived polysaccharide gels have received attention for their potential, as they are extracted from biomass, which is considered a green source that can be obtained in a sustainable way. Accordingly, this paper focuses on plant polysaccharide gels, detailing their sources, characteristics, extraction methods, main properties, and applications in the pharmaceutical and biomedical fields.

### 3.1. Developments, Characteristics, and Characterization

The dynamic polysaccharides’ nature is highly diverse, due to their nature, composition, and chemical [19] interactions. Hydrogels are produced by several gelling methods (physical, thermal, and ionic); however, chemical methods provide a controlled crosslinking, resulting in modifications, as they can affect the polymer biofunctionality [20,21,22]. According to this, we present some chemical characteristics of plant-derived polysaccharides and gelling behavior (Table 1) to better comprehend the engineered plant polysaccharide gels.

Hydrogel innovation and development are directly allied with characterization analyses, to investigate several factors, including chemical composition and behavior, thermal and mechanical properties, among others. Therefore, conventional assays for the hydrogels’ characterization consist of analyses of spectroscopy-Fourier-transform infrared spectroscopy (FTIR-chemical bonds and functions), diffractometry-X-ray (XRD-degree of crystallinity), microscopy-scanning electron (SEM-surface morphology), atomic force (position of the chemical bond functions), thermal-differential scanning calorimetry (DSC-material transitions by variation of temperature and time), thermogravimetry (mass variation for temperature increase), mechanical-(universal test-Young modulus), water swelling (quantification of water intakes), and sol–gel (hydrogel crosslinks) [37,38].

### 3.2. Cellulose

**Figure 6:**
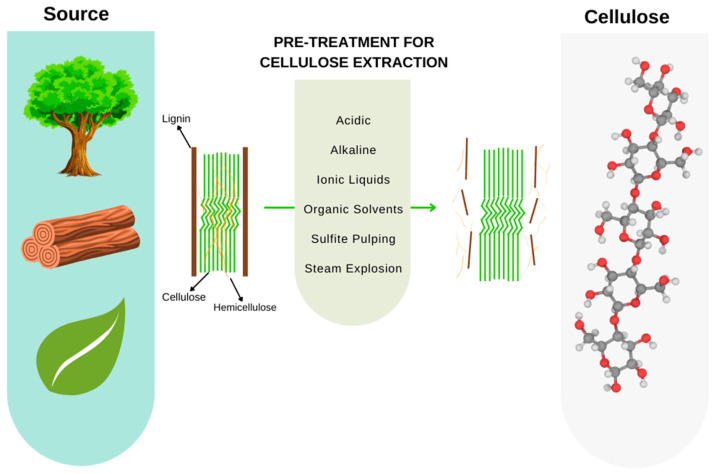
Lignocellulosic biomass: sources, extraction processes, and cellulose structure (Adapted with permission from Ref. [4]. Copyright 2015, Elsevier).

Cellulose is the most abundant polymeric material on our planet, made up of β- (1→4) glucose units. It is a linear homopolymer with extensive hydrogen bonding forces acting to promote its crystalline properties (Figure 6). There is an increasing interest in producing materials from cellulose since it is a sustainable source, with large availability, low cost, and non-edible competitiveness as regards the biorefinery concept to produce value-added cellulose-based materials.

This is a crucial ingredient for the manufacturing of various products, such as paper, textiles, membranes, biofuels, and chemicals. Its chemical structure is determined by intermolecular interactions, crosslinking reactions, chain length, and the distribution of groups along its polymeric chains [39]. Unlike synthetic polymers, cellulose-based polymers have a distinct polyfunctionality, high rigidity, sensitivity to hydrolysis, and oxidation of the chain-forming acetyl groups that determine their chemistry [40,41,42,43].

The extraordinary swelling capacity of cellulose-based gels directed its use as a superabsorbent in various applications, such as healthcare area (tampons, diapers), agriculture (soil conditioning), and biomedicine (wound dressing) [44]. Tissue engineering and pharmaceutical applications of cellulose materials have been of interest because of their biocompatibility, permeability, hydrophilicity, and non-toxic profile, which are good for controlled drug release; moreover, they mimic the extracellular matrix and have sufficient strength and flexibility to replace cartilaginous tissue [45,46]. The cellulose-based gels are adequate for drug delivery systems, because such materials swell in water, directing the water toward the drug core [47,48].

Cellulose participates in esterification, which is an equilibrium reaction that uses alcohol with an acid to form ester and water. A common cellulose-based hydrogel is carboxymethylcellulose (CMC); however, a huge variety of cellulose can be obtained via derivatization and blending [49]. The main cellulose esters are cellulose acetate, cellulose acetate phthalate, and hydroxypropyl methylcellulose phthalate. Another derivative is oxycellulose, which is produced by the oxidation of the hydroxyl group in each anhydro glucose unit of cellulose [47,50], such oxycellulose gel can control the release of encapsulated diclofenac sodium for reducing ulcerogenic activity [51] since this drug depends on the pH, in the gastrointestinal tract, to be absorbed [52].

Cellulose nanofibers are a green, biocompatible, and biodegradable material widely used in food and biomedical studies. The cellulose hydrogel can be manufactured as scaffolds for tissue engineering, and as a promising substitute for the extracellular matrix (ECM) [53]. In fact, this material showed satisfactory results as a support for liver tissue engineering, providing greater expression of liver genes, and increased hepatocytes’ functionality [54]. Cellulose nanofibers incorporated with silver nanoparticles are also present in antimicrobial and healing activity [55]. A silk/cellulose-based nanofiber is used for drug delivery of doxorubicin encapsulated [56], a drug used in chemotherapy treatment [57]. Polyethyleneimine-grafted cellulose aerogel is reported as a drug delivery vehicle for sodium salicylate, presenting pharmacokinetics closely related to the pH and temperature of the release environment [50]. Another topical application of cellulose-based hydrogel is as a drug vehicle for hydroquinone, used for inhibiting the production of melanin to prevent melasma [13,58]. Several commercial wound dressings based on CMC are available in the market by the producers Convatec, Smith and Nephew, Coloplast, and First Water [9].

A hybrid system of alginate-gelatin and cellulose nanocrystals (CNC) has been studied which promotes cell or biomolecules delivery for tissue engineering applications [59]. A hybrid hydrogel (CNC and thermosensitive poly (N-isopropyl acrylamide)) enhanced the mechanical properties of the acrylamide gel and reduced brittleness during stress, in addition to the possibility of antibiotic incorporation [50,60,61,62,63]. CNC gel treated with carboxymethylation and periodate oxidation, followed by 3D printing resulted in a scaffold that can act as wound dressing since it inhibited bacterial growth [64].

Cellulose hydrogels can be also used as injectable implants since they are biocompatible, biodegradable, mechanical adaptable, with great porosity, characteristics that favor drug administration [65]. This approach has been investigated for CMC as injectable bone cement, encapsulated with Ag^+^ nanoparticles to enhance the bactericidal activity in implants, minimizing the risk of rejection [66]. A propranolol-CMC bionanocomposite hydrogel granules showed a sustained release pattern in gastrointestinal conditions [67]. CMC has also application as bioink for 3D printing in tissue engineering applications, such processing technique results in scaffolds with tunable shapes and properties, in addition to customizable pieces for regenerative medicine [68].

### 3.3. Hemicelluloses

**Figure 7:**
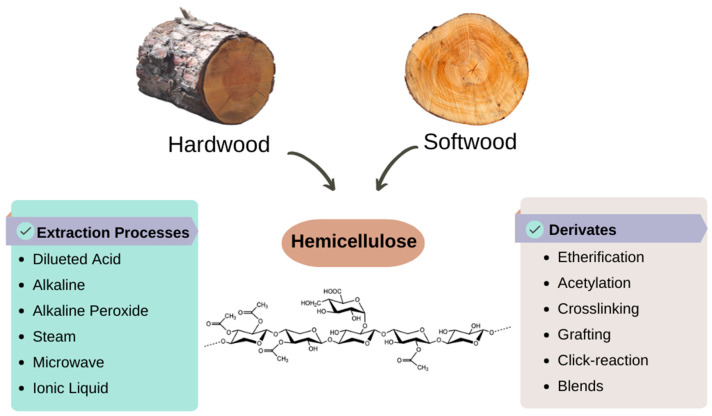
Hemicelluloses: Source from hardwoods, general structure, extraction routes, and derivatives mechanisms (Adapted with permission from Ref. [71]. Copyright 2017, Wiley).

Hemicelluloses are a branched copolymer composed of pentoses and hexoses-xylans, mannans, β-glucans, and xyloglucans, they have shorter polymeric chains than cellulose, and are mostly available in hardwoods [69,70] (Figure 7). Some compositional differentiation can be found in the hemicelluloses, regarding their origin, since hardwood-derived hemicellulose is rich in acetylated xylan, while softwood is less acetylated [71].

Different methods for hemicelluloses extraction facilitate their recovery into the liquid phase; for instance, water extraction (subcritical water, autohydrolysis), and dilute-acid extraction [71,72]. Autohydrolysis is an environmentally friendly process, because the lignocellulosic biomass is pre-treated with hot water, promoting the extraction of hemicelluloses into the liquid phase, and enabling the hemicelluloses valuation [73]. High temperature and pressure operational conditions are important factors in hydrothermal processes, because such variables help the water autoionization and disintegration of the lignocellulosic matrix [74]. These conditions expand the cellulose’s surface area, promoting hemicellulose depolymerization, and lignin re-localization [73,75]. Additionally, an acidifier medium (dilute-acid) might boost hemicelluloses recovery compared to simple autohydrolysis [76,77]. As an example, subcritical water (100–374 °C; 0.1–22 MPa) has an acidic nature (increment in [H_3_O^+^]) that intensifies the hydrolytic reactions of lignocellulosic components [73]. Steam explosion occurs at temperatures between 210 and 290 °C, and high pressures (20–50 bar), in a short period, as the condensed steam breaks the inter and intramolecular linkages from the lignocellulosic components [74].

There is increasing interest in adding value to the hemicelluloses since they can act as a raw material for several products, and usually, it is wasted in the lignocellulosic pre-treatment as part of black liquor that is burned to produce energy [71]. Among these, the rich hydroxyl and carboxylic structure of the hemicelluloses allow several chemical derivatizations without loss of inner properties regarding biocompatibility and biodegradability, which is highly desirable for drug delivery materials, in addition to the fact that the hemicelluloses are reported as anti-cancer and anti-inflammatory components [25,78]. Hemicellulose derivatization might also produce biopolyesters [79].

The hemicellulose-based gel is a new field in pharmaceutical applications, being studied as a drug delivery system, with mostly acetylsalicylic acid and theophylline as model drugs [80]. Hemicellulose-gels can be used as wound dressings, as some studies evaluated the ability of hemicellulose to prevent infections from particulates and germs in the wounds, in addition to exploring their capacity to accelerate the regeneration of human skin [71,81]. Its blend with gelatin, showed targeted antibiotic (gentamicin) in the wound, promoting faster healing, meaning an improvement in the patient’s life [82]. Interestingly, a hemicellulose hydrogel incorporated with magnetic Fe_2_O_3_ is attracted to metallic nanoparticles (Au, Pt, Pd), in a way that this material works as a metal detoxifier [83]. Complementarily, hemicellulose-based hydrogel with polyamidoamine has heavy metal (Pb, Ni, Zn, Pd) adsorption properties [84].

### 3.4. Lignin

Lignocellulosic biomass provides lignin fraction, which is surrounded by cellulose and hemicelluloses molecules. The sources for lignin are the same as the wood-cellulose, including agricultural waste. The lignin composition and content depend on several factors, such as plant type, maturation, piece of plant, cultivation parameters, and the extraction processes, etc. [85]. Lignin acts as plant-cell cement that helps the mechanical strength of the plant, constituting between 15 and 40% of its dry mass [86].

**Figure 8:**
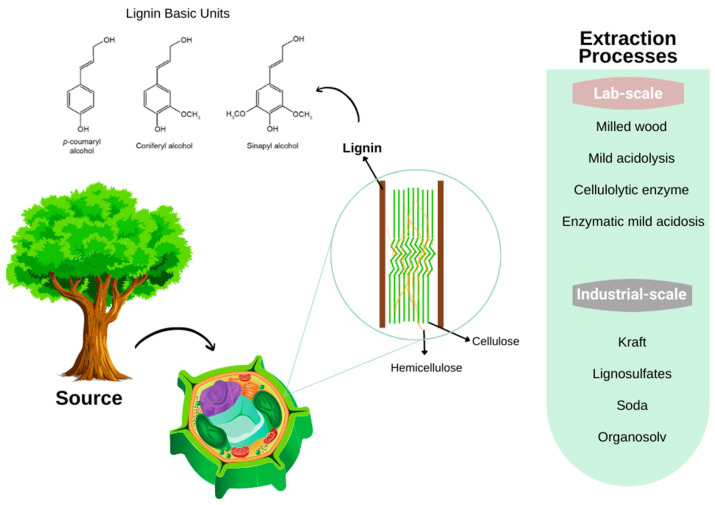
Lignin: structure and extraction processes at different scales (Adapted with permission from Ref. [93]. Copyright 2020, Springer).

The complexity of the polyphenolic lignin structure (Figure 8) makes it difficult to standardize its properties, which are constituted of highly aromatic polysaccharides. Moreover, lignin is constituted of monolignols, specifically p-hydroxyphenyl (H), guaiacyl (G), and syringyl (S) units, its linkages and ramified groups provide molecular structure diversification. Parallel to the cellulose and hemicelluloses extraction, lignin isolation also derives from thermal, mechanical, chemical, and biological treatments [87,88].

For the lignin applications in the pharmaceutical or biomedical areas, its extraction process needs to be solvent and sulfur-free, yielding high-value lignin. Organosolv extracts lignin with an organic solvent, such as methanol or ethanol, at high temperatures and pressure. Hydrothermal uses hot water for the separation of lignin, producing almost a native lignin that is great for producing an aerogel [89] that can be applied in tissue engineering and regenerative medicine [27,90].

An example of a lignin-derived polymer is polyurethane (PU), which polyol-rich structure of lignin act as a monomer in the PU synthesis. Other resins based on lignin are also available, such as epoxy, and in addition to it, lignin can act as a carbon fiber used as fillers in polymeric composites [91]. Unusual applications of lignin-based materials are binders, separators, and electrolytes (anodes and cathodes) for rechargeable batteries as lignin is itself electrically active [92].

Lignin-based gels have antioxidant and antimicrobial properties, UV absorbing capabilities, and low cytotoxicity, due to their chemical groups (phenols), properties that are highly desired for biomedical and pharmaceutical applications [94,95,96]. Additionally, the UV protection of lignin-based materials, their photostability and radical scavenging stimulated their use in sunscreen formulations, as nanoparticles [97], which enhance the sun protection factor (SPF) and water resistance due to its adhesiveness [98]. Thanks to these properties and biological activities, lignin has been combined with other polymers (e.g., chitosan, poly (vinyl alcohol) (PVA), alginate, cellulose) to produce hydrogels for wound-healing applications [89]. Additionally, different formulations (encapsulated, emulsified) and processing techniques (3D-printing, electrospinning) of lignin-based materials, have expanded their applications in the biomedical area [99].

Moreover, lignin can increase the mechanical strength of numerous processed biomaterials, for this reason it is studied as a green material for health areas, particularly in drug delivery systems and wound dressing, because it has a great absorbing capacity, with the capability to remove undesirable metabolites from the tissues, regularly delivering incorporated drugs to the affected area, in addition to the other aforementioned qualities. 

A superabsorbent hydrogel based on cellulose and lignin hydrogel can be obtained by dissolving cellulose in an alkali solution with further mixing with lignin, using epichlorohydrin as chemical crosslinking [100]. The lignin copolymerization with ethylene glycol and methyl vinyl ether-co-maleic acid via esterification resulted in a hydrogel that can be used as medical coatings [101]. Oxygenated lignin copolymerized with alkylene oxide and alkyl ether methacrylate is a versatile hydrogel, with diversified applications in biomedical and personal care, as a support for an active agent or controlled drug release. It can also reconstitute damaged parts of bodies (e.g., wounds, joints), and in cosmetics, it can be used as a hair gel product. Ag-lignin hydrogel has adhesive properties that might be a potential surgical glue, in addition to which, the highly oxygenated chemical groups can reduce Ag^+^ producing in situ bactericidal agent [102]. Lignin-incorporated nanogel composites showed efficiency to speed up the healing of wounds, being confirmed with in vitro and in vivo studies, reinforcing the safety of lignin-based materials in contact with injured skin [103]. Such properties also attracted the attention for transcutaneous drug delivery in materials containing lignin, as it presents mucoadhesiveness, being a candidate material for electromagnetic response, local anesthesia, dermatological, and chronic wound treatments [104]. The UV protection of lignin-based material had been explored in the textile area to produce clothes that lower the sun’s damage to the skin [105].

### 3.5. Inulin

Inulin is an oligosaccharide discovered in the 19th century and found in a wide range of plant species as an energy storage component [106]. Usually, inulin is extracted from chicory roots and Jerusalem artichoke that contains up to 20% w/w of inulin, in an energy-consumption extraction process. Other assisted extraction procedures use microwave, ultrasound, pulse-electric field, and supercritical fluid. An alternative route to produce inulin involves an enzymatic pathway with inulosucrase [107,108].

**Figure 9:**
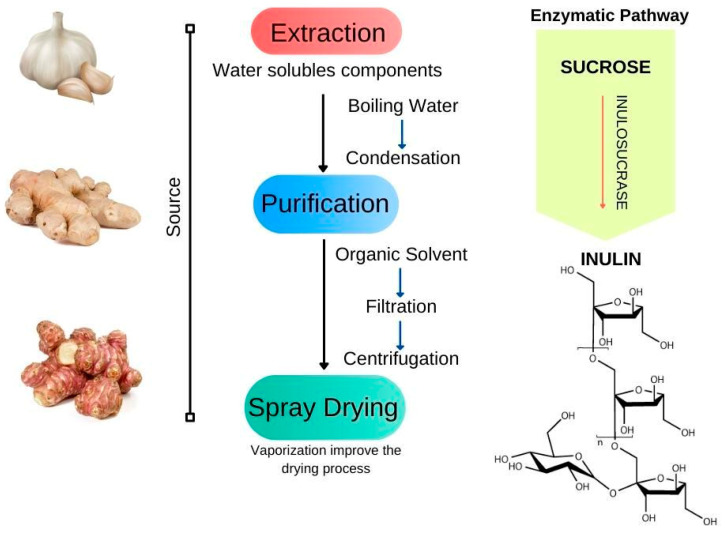
Inulin: sources and obtaining methods.

Considered a versatile ingredient due to its health benefits, inulin is considered to be a fermentable oligo-, di, monosaccharides, and polyols, belonging to the group of carbohydrates digested in the colon that promote water absorption, controlling constipation processes, and other related diseases [107]. It is a poorly soluble polymer and has a high molecular weight (6179 g.mol^−1^) compared to other mono and disaccharides (<500 g.mol^−1^), thus it presents high glass transition and melting temperature, but it has molecular flexibility due to its structure (2 → 1) d-fructosyl [109,110] (Figure 9). The inulin gel’s three-dimensional structure has compatible physiological activity, biodegradability, and compatibility, which makes inulin-based gels an ideal material for drug delivery as they swell physiological fluids [111].

In the food sector, the inulin acts as a texture enhancer, a non-digestible fiber used in bakery products, and a prebiotic compound in dairy products, in addition to being a low-calorie product used to substitute sugar and fat ingredients. Inulin-based hydrogels provide promising delivery systems, mainly in the treatment of intestinal diseases, since inulin is only degraded by bacteria present in the colon [112,113,114,115]. Exploring this characteristic of inulin-based hydrogels, a nanocomposite of inulin and silica nanoparticles was formulated, intended to be produced to be a gut bacteria nanocarrier system to reach the intestinal microbiome [116]. Inulin-bioconjugated β-cyclodextrin can deliver hydrophobic drugs used in colon diseases treatment, preventing early absorption that can cause collateral effects on patients [117]. Other works involving inulin-based hydrogels for target colon drug delivery can be found in the review written by Giri et al. (2021) [118]. Biomedical application of inulin-coated Fe_3_O_4_ nanoparticles includes their use to enhance magnetic resonance imaging for diagnostic liver diseases [119]. Chitosan and aldehyde functionalized fructan forms an injectable hydrogel that can encapsulate dopamine, with an adequate physiological response (degradability, cytocompatibility) [120].

### 3.6. Pectin

Pectin is a plant-derived, naturally occurring anionic polysaccharide polymer that is present in the plant cell wall, mostly formed by α-1,4-linked-D-galacturonic acid and some galacturonan-derivatives (homo-, rhamno-, xylo-) [121]. This component is present in plants and has a structural function, predominantly as a mechanical strengthener and cell adhesion agent usually found associated with lignocellulosic compounds [122]. Due to the high complexity of its structure, its chemical composition is still under debate, but the literature describes its role in plant growth and development, as well as in fruit ripening and fiber processing.

**Figure 10:**
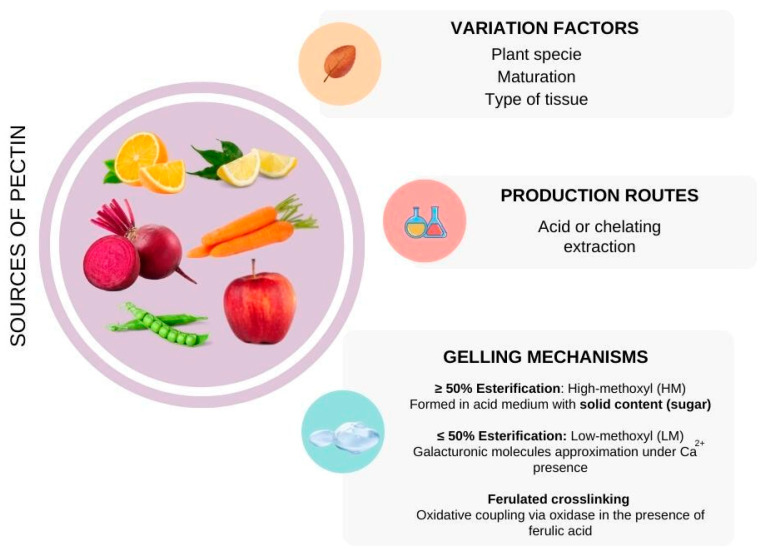
Pectin: sources, extraction method, and gelling mechanisms.

Various fruit and vegetables are sources of pectin (Figure 10), especially the citruses, apple pomace, and sugar beet [123]. The extraction procedure to produce pectin commonly involves an acidic or basic medium in the presence of enzymes or chelating agents, and depending on the esterification degree of the pectin there is a most suitable method. The raw material should suffer acidic hydrolysis under heating to produce high-methoxylated (HM) pectin, otherwise, basic hydrolysis provides a low-methoxylated (LM) pectin. After hydrolysis, the plant pulp is pressed or centrifuged to separate the pectin from the liquid phase; however, if the intention is to obtain the pectin powder, it must suffer precipitation in alcohol to remove impurities, followed by drying and milling.

This biopolymer is of great interest to the food and pharmaceutical industries due to its rheology modifier function, with high gelling/thickening property, ability to form aqueous gels, in addition to its biocompatibility and non-toxicity [32,124]. In the food and beverage industry, pectin plays an important role in the sweet sector, acting as a gelling component in fruit concentrates (jam, syrup, yogurt), preventing solid flotation and enhancing the texture. In the pharmacological scenario, pectin has several functions as a cholesterol reducer, anti-poisoning metallic cations (“egg-box model”), and satiety enhancer, among others [125]. Additionally, the pectin gels can be used as emulsifier agents to improve cosmetic emulsion stability and delivery of drugs and active agents into the skin [126].

A pectin/honey hydrogel had in vitro and in vivo efficiency against *S. aureus* and *E. coli* strains, and had good results in fluid uptake, non-toxicity, and good swelling capacity, which is relevant in reducing the risk of wound dehydration during the healing process [127]. Pectin hydrogel enriched with allantoin (moisturizer) promoted a 25% increase in speed of healing in rats with injured skin [128]. Pectin-Fe^3+^/hydrophobic polyacrylamide dual-physically crosslinked hydrogels presented adjustable mechanical properties with satisfactory anti-fatigue and time-dependent self-healing capabilities. Moreover, the mechanical properties, the in vitro assays demonstrated cell infiltration in the hydrogels, with cell adhesion and proliferation, demonstrating promising application in tissue engineering [129]. Low methoxyl pectin/gelatin/carboxymethyl cellulose ternary film showed promising antimicrobial properties to be used as dressings for dermatological use [130].

A pectin/polyacrylamide hydrogel facilitated the colon-targeted budesonide delivery for the treatment of ulcerative colitis [131]. Copolymeric pectin hydrogels may help the controlled delivery of galantamine used in patients with Alzheimer’s disease, showing enhanced galantamine loading with the pectin present in the formulation, slowing its release [132]. N-succinyl chitosan / oxidized pectin is a hydrogel that can be injectable since it is shear-thinning, acting as a scaffold for drug release, and showing anti-hemolytic properties. A derivate hydrogel from pectin, low methoxy pectin, forms a composite with sodium alginate and nanocellulose fibers; that presented effective clindamycin delivery since this pectin-derived gel showed low cytotoxicity for HaCaT cells [119]. Microspheres composed of pectin/magnetite present magnetic sensibility, which when combined with anticancer agents can adequately help chemotherapy treatments as a drug vehicle system [133]. Pectin-based gels are customizable and can be formulated for target drug delivery systems (nasal, oral, ocular, dermal, vaginal), as they are a safe material with properties that may produce the smart release of drugs [134].

### 3.7. Starch

A sort of plant resource is rich in starch, including cereals and legumes, being a storage carbohydrate found in grains, tubers, and roots, consumed by humans, such as corn, potatoes, pine nuts, cassava, wheat, etc. [134,135]. Starch is a sustainable alternative because it allows the synthesis of polymers from renewable sources, is eco-friendly, and is widely available in nature [134].

**Figure 11:**
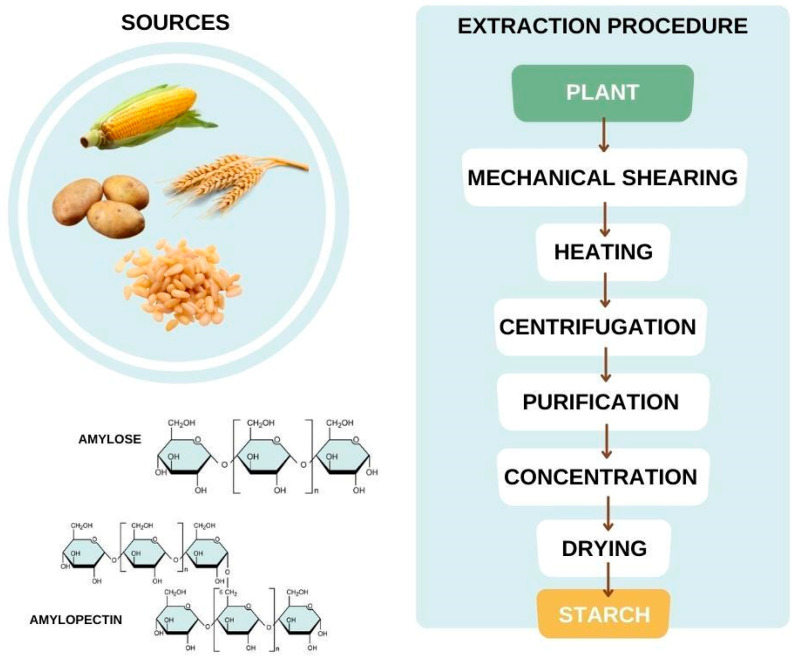
Starch: Sources, chemical composition, and extraction procedure (Adapted with permission from Ref. [141]. Copyright 2016, Elsevier).

The starch structure (Figure 11) consists of amylopectin and amylose units that form a high molar mass polymer with hydrophilic behavior, both consisting of α (1,4) glucopyranose, while amylopectin is linked with α (1,6) bonds, with the last one with the higher degree of polymerization [136,137,138]. As amylopectin is a smaller molecule, it allows a higher organization/crystallization, while the amylose confers amorphous regions in the starch [139]. The amylose and amylopectin contents in the starch may vary according to the plant species and maturation [140].

After the starch granule is exposed, the purification step occurs under an inert atmosphere with polar organic solvents; usually, one where amylose is insoluble, followed by precipitation sequences according to the desired purity level.

The starch gelatinization process occurs under hot water swelling [142], causing crystallinity reduction with irreversible structure expansion, producing a polyhydroxy polymer [143]. This gelling behavior is explored in the food industry for viscosity enhancement in jams, jellies, soups, creamy powders, and sauces; it is also appealing since it is gluten-free and feels firm in the mouth, properties which can also be achieved with different biopolymer formulations with starch [144,145].

Such green material has also several non-food applications, for example, food packaging, health care, cosmetics, pharmaceuticals, textiles, and building, among others [146,147]. Currently, modified starch is applied in adhesives and packaging, mainly for the pharmaceutical and cosmetics sectors, as its properties can be enhanced to tailor its final application [148].

In the pharmaceutical industry, several classes of amide gels are used in wound healing. One example is the bioactive sodium carboxymethylated starch hydrogels containing copper oxide nanoparticles that presented antibacterial and antioxidant activities, with healing potential according to the in vivo study [149]. Carboxymethylation produces a modified starch with greater water solubility, widening its uses, but is popularly employed as an excipient in tablet formulations, due to its low cost, intrinsic biodegradability, and non-toxicity [150]. Additionally, in the pharmaceutical sector, Contramid^®^ is a commercial hydroxypropyl and crosslinked high-amylose starch used as an excipient for oral drugs to slow the drug release [151].

The type of starch presented in tablets can influence the drug release, mostly regarding the crystallinity aspect, which has proportional resistance to enzymatic systems; another aspect observed was a faster disintegration and drug release for starch with a higher crosslinking degree [152]. A slowly digestible starch, which is also resistant, is ideal for oral colon-specific drug delivery, as it passes intact through the digestive system to disintegrate in the intestine [153,154].

Microfibrillated cellulose films from yerba mate extract and corn starch also presented a wound-healing effect [155]. Composite gels based on gelatin and rice starch loaded with lysozyme and green tea polyphenols showed promising antimicrobial, antioxidant, bioactive, and sterilizing properties for application in the biomedicine and pharmaceutical sectors [156]. Pinion starch gel presented great antioxidant activities for pharmaceutical and food applications [157]. A strontium crosslinked starch hydrogel has potential as a wound dressing material because, in addition to the good characteristics of starch, such material can be injectable, presenting self-healing and adhesiveness properties to protect the damaged tissue [158]. Interestingly, a composite-based starch-containing nanocellulose and carrageenan has hemostatic properties for blood clotting and stops hemorrhaging [159].

Starch has an instigating chemistry for the development of materials, this chemical versatility produces polymers with different properties that can be tunable for several biomedical applications according to the desired final function [160].

### 3.8. Guar Gum

Guar gum, also known as cyamopsis, guaran, guyan, guarina, or glucotard, is a natural water-soluble polysaccharide obtained from the guar plant, specifically at guar’s bean seed, guar gum has the biological function to be a reserve nutrient [161]. It is a nonionic branched polymer with a high molar mass, composed of mannose and galactose molecules. At a low temperature, it presents a viscous solution, with stability at pH 5–7; however, at pH values of 6–9 it has the maximum viscosity.

The gum extraction from the guar plant consists of physical separation processes after its beans are dried, to better collect the endosperm, followed by grinding, sieving, and dehusking [94,162], which finally flaking and milling produce the guar gum powder with the desirable characteristics according to the process parameter.

**Figure 12:**
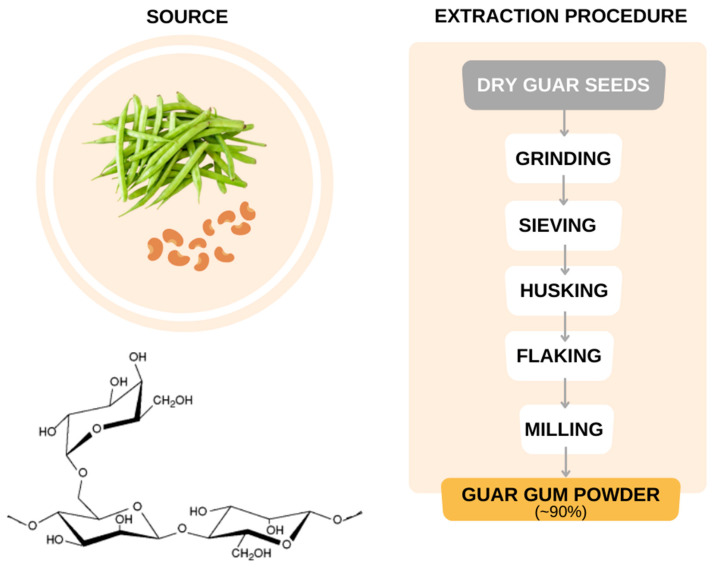
Guar gum: origin, extraction procedure, and chemical structure (Adapted with permission from Ref. [161]. Copyright 2016, Elsevier.

This polymer is very used in the development of hydrogels because this polysaccharide disperses in water. Its hydroxyl groups mainly from (1,6)-linked α-galactose side chain to the linear (1–4)-β-D-mannose structure interacts with water leading to entanglement (steric effect) of the intermolecular chain leading to the gel behavior [161] (Figure 12). Therefore, rich galactose regions are more hydrosoluble than poorer ones, as the last ones present higher crystallinity and lower solubility. An unusual function of guar gum is its dispersant effect on organic solvents containing hydroxyl groups (-OH, -COOH) due to its exposure to hydroxyls, which also act as mineral coagulants [134].

Several uses for the guar gum can be found, i.e., human consumption, as dietary fiber; pharmacological component, as tablet binding agent and viscosifying syrup; personal care, cosmetics emulsifier; stabilizing agent for paints and coatings; agriculture, superabsorbent polymer, bioremediation agent [162].

The antimicrobial activity of hydrogels based on guar gum and arabinoxylan (extracted from Plantago bark seeds), using tetraethylorthosilicate as a crosslinking agent, has been investigated [163]. The guar gum/arabinoxylan hydrogels showed antimicrobial properties against strains of *P. aeruginosa* (Gram-negative) and *S. aureus* (Gram-positive), nontoxic cell behavior, with a satisfactory release profile. The results presented a porous morphology, with interconnected pores attributed to the increase in the crosslinking and dilation of the hydrogel confirmed by electron microscopy. Hydrophobically modified guar gum loaded with antibiotics (gentamicin, amoxicillin) also presented bactericidal (*Staphylococcus aureus*, *Escherichia coli*) and antifungal (*Candida albicans*) activity, favoring its use as wound dressing [164]. Crosslinked guar gum-g-poly (acrylic acid-co-acrylonitrile) is efficient in a medium with different pH to deliver thymoquinone and inhibit inflammation [165]. Another pH-responsive guar gum-based gel, a blend with sodium alginate and polyvinyl alcohol, showed a good release profile of verapamil hydrochloride [166], confirming the robustness of guar gum-based gels for drug delivery. As injectable material, chitosan and guar gum-based hydrogel is a potential system for target chemotherapeutic agent (doxorubicin) delivery, in which the drug effectiveness at the tumoral pH medium is fundamental to killing cancer cells [167].

Beyond the guar gum itself, its derivatives (hydroxymethyl-, hydroxypropyl-, carboxymethyl-, sulfated-, etc.) are widely employed in target colon drug delivery [168,169]. Several works developed guar gum-based hydrogels incorporated with silver nanoparticles for biomedical applications due to its bactericidal behavior [170].

Gums of natural origin are widely used in the pharmaceutical industry in the development of liquid, topical and oral products. Cashew-gum is a plant polysaccharide that has enormous potential for applications as a hydrogel in the medical-pharmaceutical areas, this gum is a branched acid heteropolysaccharide composed of galactose, glucose, glucuronic acid, arabinose, rhamnose, and mannose. Usually, its production occurs in the epithelial cells present in the tree’s bark in response to mechanical stimuli or pathogen attacks [171,172,173]. Cashew gum and chitosan (1:4) hydrogel could be used as a material for skin lesions treatment, because it modulated the inflammatory process, promoted better wound contraction, increased collagen production, extinguished necrosis, and induced early epithelization in rats [174]. Studies have associated cashew gum and CMC hydrogel with silver nanoparticles (AgNPs) to promote greater stabilization of the colloidal system and also to improve biocompatibility. The antimicrobial activity of cashew gum/CMC/AgNPs hydrogels was evaluated in two models, the first in vitro model against strains of *S. aureus* and *P. auriginosa*, and the second in vivo model using rats. Both experiments resulted in bacterial inhibition and wound healing in the animals, characteristics that detach the potential for wound-healing applications of the cashew gum/CMC/AgNPs hydrogel [175].

## 4. Future Perspectives

The search for sustainable, biodegradable, biocompatible, biologically safe materials, with mechanical stability and adjustable functionality, encourages the development of hydrogels based on polysaccharides [176,177]. In this context, hydrogels from plant sources deserve special attention since their potential has not yet been fully explored in clinical or industrial areas [176].

Currently, hydrogels are used in the manufacture of biomedical products, such as contact lenses, tissue engineering supports, drug delivery systems, wound dressings, sensors, and bioelectrode devices, among others [178,179]. Exploring smart hydrogels capable of managing drugs and bioactive agents, particularly anti-cancer drugs, is challenging.

To increase the attractiveness and applicability of plant polysaccharide gels, some technical challenges still need to be addressed, regarding their compositional uniformity, mechanical strength/tenacity, and stability [139,180]. Some scaffold architectures to produce wound dressings can be processed using spinning techniques (electrospinning, rotary jet spinning) or 3D biomanufacturing, resulting in hydrogel structures that mimic the extracellular matrix, enhancing cell adhesion and proliferation, in addition to fomenting a controlled drug release.

Talking about the commercial application of hydrogels’ products, several products are formulated with plant-based polysaccharide gels (cosmetics, food, drugs, etc.). However, some challenges must be overcome for noble applications (biomedical and pharmaceutical areas), such as the use of non-toxic solvents or reagents during their synthesis, which is still a problem depending on its application, specifically those that involve direct contact with humans. Future research should be devoted to the selection of safe and energy-efficient solvents, and reagents in the hydrogels’ synthesis processes to meet these requirements [176,179]. Polysaccharide hydrogels from plants have a complex composition/structure, which might difficult their standardization of properties, but chemistry solutions have emerged to improve the gel’s properties. Thus, chemical modifications (derivatization) can expand their versatility, including blending, copolymerization, and addition of fillers that can confer mechanical reinforcement and the ability to respond to external stimuli (pH-responsive, magnetic responsive, among others).

At present, there are limitations to scaling the production processes of hydrogels, leaving the laboratory scale for an industrial one, this bridge is important for the affirmation of the product at a commercial level. Meanwhile, the intensification of development to obtain hydrogels with innovative properties, homogeneous composition, diameters, thicknesses, pore sizes, and density, for efficient biomedical and health applications, is compelling [8,178].

As previously described, the development and application of hydrogels in different areas need greater interaction between the research areas, which allows us to conclude that the combination of multidisciplinary researchers is fundamental for the advancement of these materials and their applications.

## 5. Conclusions

The use of plant-derived polymers in the pharmaceutical and biomedical industry has grown exponentially in recent decades due to their low cost, better biocompatibility, and degradability. The use of these polymers in the development of hydrogels represents great advances in the production of more renewable products. Thus, several studies showed satisfactory results and advances in the development of bioproducts using plant polysaccharides, such as lignocellulosic biomass, gums, inulin, pectin, and starch. In drug delivery, they can promote a controlled drug release, improving the bioactive effectiveness. As wound dressing, biogels have antimicrobial properties, acting as support for cells to adhere and grow. Moreover, their great fluid swelling capacity resulted in fast wound healing in several studies, since it protects the wound from external contamination and absorbs exudates. Notoriously, such monomers (plant-derived polysaccharides) are metabolized by the human body, representing a safe source for health applications. Their mechanical strength can also be enhanced with cellulose nanocrystals, expanding their applications that urge this property, especially in the tissue engineering field. The compositional versatility of biogels allows several routes to explore their properties and applications, being an attractive theme for study by the scientific community.

## Figures and Tables

**Table 1:** Plant-derived polysaccharides, their types, chemical characteristics, and gelling behavior.

Polysaccharide	Chemical Characteristics	Gelling Behavior	References
Cellulose	Easy chemical modification; high degree of crystallinity; adequate mechanical properties, and great specific surface area	It forms a semi-interpenetrating polymer network	[23,24]
Hemicelluloses	A huge amount of hydroxyl groups allows chemical modifications	It presents film-forming properties due to gelation, with satisfactory mechanical properties	[25,26]
Lignin	Rich in phenolic and aliphatic hydroxyl groups that confer chemical versatility	It can form a continuous phase by gelation, with particulate-filled polymer networks	[27,28]
Inulin	Branched fructosyl units	Typically, inulin properties highly depend on their degree of polymerization	[29,30]
Pectin	Rich in carboxylate units (methyl esters)	The degree of esterification controls its gelling mechanism	[31,32]
Starch	It is formed by rich oxygenated units (amylose and amylopectin)	First, the starch is modified by physicochemical routes, and then, using hot water, the starch breaks down and swells the amorphous and semi-crystalline regions; smart starch gels can be obtained since it responds to stimulus	[33,34]
Guar Gum	Mainly composed of D-mannopyranose unit, with various hydroxyl groups	Usually involves slow gelling process time (low productivity)	[35,36]

## Data Availability

Not applicable.
